# Characteristics of antioxidant capacity and metabolomics analysis of flavonoids in the bran layer of green glutinous rice (*Oryza sativa* L. var. Glutinosa Matsum)

**DOI:** 10.1038/s41598-023-43466-3

**Published:** 2023-09-29

**Authors:** Chenggang Liang, Zhixiu Guan, Kesu Wei, Wujuan Yu, Li Wang, Xuling Chen, Yan Wang

**Affiliations:** 1 Institution of Plant Genetics and Breeding, Guizhou Normal University, Guiyang, 550001 China; 2 Guizhou Academy of Tobacco Science, Guiyang, 550003 China

**Keywords:** Computational biology and bioinformatics, Plant sciences, Health care

## Abstract

Green glutinous rice is a unique genetic germplasm that has yet to be adequately studied. This study investigated antioxidant capacity and flavonoid metabolites in the bran layer of green glutinous rice (LvH) compared to purple (HeiH), red (HongH) and white (GJG) varieties. The results showed that LvH bran had significantly higher content of total flavonoids and anthocyanin than that of HongH (1.91-fold and 4.34-fold) and GJG (2.45-fold and 13.30-fold). LvH bran also showed significantly higher levels of vitamin B1 and vitamin E than that of HeiH (1.94-fold and 1.15-fold) and HongH (1.22-fold and 1.13-fold), indicating that green glutinous rice bran was rich in bioactive components. LvH bran showed significantly lower IC_50_ values for scavenging DPPH and ATBS radicals than GJG and even significantly lower IC_50_ value for scavenging DPPH radicals than HongH, highlighting its potential as an effective source of antioxidants. LvH bran had significantly different downstream metabolite synthesis in the flavonoid pathway compared to HeiH, HongH, and GJG, with 40, 26, and 22 different metabolites, 23, 20, and 33 up-regulated differentially expressed metabolites (DEMs), and 73, 50, and 13 down-regulated DEMs, respectively. Of the 139 flavonoid metabolites identified in colored rice bran, 26 metabolites showed significant positive correlation with both ABTS and DPPH radical scavenging capacity. Typically, quercetin derivatives showed potential for evaluating the antioxidant capacity of colored rice bran. These findings offer valuable insights into the antioxidant properties of green glutinous rice bran and provide references for better understanding of flavonoid metabolites in different colored rice bran.

## Introduction

The bran layer of rice (*Oryza sativa* L.), which consists of the pericarp, aleurone, subaleurone and germ, accounts for approximately 5–8% of brown rice and is the primary by-product of rice milling^1^. Rice bran is rich in bioactive compounds that have potent antioxidant, antimicrobial, cancer chemopreventive, antidiabetic, and hypolipidemic properties^2,3^. However, during the conventional rice milling process, the bran layer is pulverized, thereby resulting in a significant loss of the physiologically active components, such as flavonoids, anthocyanins, and proanthocyanidins metabolites^4–6^. Recently, consumption of unpolished rice is becoming increasingly popular for maximum health benefits. In addition, rice bran is also utilized to produce foods like flour and bread^7,8^.

Flavonoids, which consist of C_6_–C_3_–C_6_ rings, are the most abundant polyphenolic compounds and are mainly stored in the bran layer of rice^9^. Flavonoids, classified into flavanols, flavones, flavonols, flavanones, isoflavones, and anthocyanidins, are strongly associated with antioxidant properties^4,5,10–12^. Compared to trolox, an α-tocopherol analogue, the flavonoids with multiple hydroxyl substitutions have several times stronger antiperoxyl radical activities^13^. It is well known that naturally occurring black (deep purple), purple, and red rice germplasms contain higher physiologically active components than white rice, especially flavonoid metabolites like anthocyanins, proanthocyanidins, flavonols, and flavonoids^14–17^. Among these, water-soluble anthocyanins have been identified as the most conserved class of flavonoids after cooking by using different colored rice kernels (black, purple, and red) as materials^18^. Furthermore, it is noteworthy that there are commercial benefits from the extraction of antioxidants through the recovery of flavonoids from pigmented rice bran^19–21^. Recently, various techniques have been investigated to enhance the bioactivity of rice bran by liberating the bound form of flavonoids, including physical and biological enzymatic methods^22–24^.

Glutinous rice (*Oryza sativa* L. var. *glutinosa* Matsum), which belongs to the *Gramineae* family, is extensively cultivated and consumed worldwide^25^. It has been reported that glutinous purple rice retains more anthocyanins than non-glutinous purple rice^26^. Kam Sweet Rice (KSR) is considered a specialty glutinous rice by the Food and Agriculture Organization of the United Nations, and it has been cultivated for over two thousand years in the mountainous regions of southeastern Guizhou Province^27^. In China, KSR is renowned as ‘Xianghenuo’ for its fantastic aroma, superior quality, and well-recognized taste^28^. Consequently, despite its high price, which is about ten times that of ordinary glutinous rice, the demand for KSR exceeds supply in the market^28,29^. KSR varieties are excellent germplasm resources for the improvement and utilization of rice landraces^30^. Unfortunately, the number of KSR varieties has declined significantly over the past seven decades, and many varieties have become extinct due to inadequate productivity^28–30^. Through extensive germplasm resource surveys, more than one hundred traditional KSR germplasm resources, including many colored germplasm, have been collected^29,30^.

Colored glutinous rice has recently attracted attention for its physiologically active components and antioxidant capacity^6,14–17^. Metabolomics technology has been increasingly applied to evaluate the quality of glutinous rice and food processing^6,16–18^. For example, in a comparative analysis, 281, 305, 241, 267 and 265 DEMs are identified in the glutinous/white versus black, glutinous/white versus red, and red versus black comparison groups, respectively, through widely targeted metabolomics^31^. A total of 390 DEMs are detected between purple glutinous rice and white glutinous rice, and 6 candidate metabolite markers are identified for antioxidant capacity screening based on LC–MS metabolomics^16^. However, green rice remains a rare germplasm worldwide, and further research is needed to investigate its flavonoid components and antioxidant properties require^32^. Therefore, this study aimed to investigate the differences in total anthocyanin content (TAC), total flavonoid content (TFC), 1,1-diphenyl-2-picrylhydrazyl (DPPH) radical scavenging capacity, 2,2′-azino-bis(3-ethylbenzothiazoline-6-sulfonic acid) (ABTS) radical scavenging capacity, and flavonoid metabolites in the bran layer of green variety compared to purple, red, and white varieties of KSR. These findings provide a basis for further understanding of the antioxidant capacity and flavonoid metabolites of bran in green glutinous rice and its potential health benefits.

## Results

### Phenotype and antioxidant capacity

**Figure 1:**
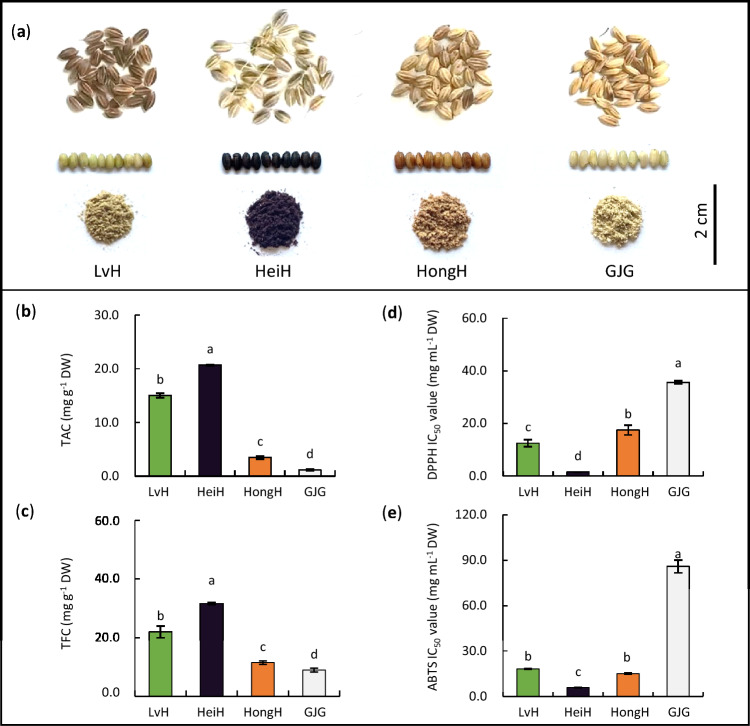
Phenotype (**a**), TAC (**b**), TFC (**c**), DPPH IC_50_ value (**d**), and ABTS IC_50_ value (**e**) in the bran of LvH, HeiH, HongH and GJG varieties. *TAC* total anthocyanin content, *TFC* total flavonoid content, *DPPH* 1-diphenyl-2-picrylhydrazyl radical scavenging capacity, *ABTS* 2′-azino-bis(3-ethylbenzothiazoline-6-sulfonic acid) radical scavenging capacity. Different lowercase letters indicate significant differences at *p* < 0.05.

The phenotypic characteristics of the grain, kernel and bran of glutinous rice varieties are presented in Fig. 1a. LvH showed a light green kernel with taupe grain, which differed from HeiH (deep purple), HongH (light red), and GJG (creamy white). LvH bran also had significantly higher TAC (15.03 mg g^−1^ DW) than HongH bran (4.34-fold) and GJG bran (13.30-fold), but significantly lower than HeiH bran (0.71-fold) (Fig. 1b). LvH bran had significantly higher TFC compared to HongH bran (1.91-fold) and GJG bran (2.45-fold), but significantly lower than HeiH bran (0.69-fold) (Fig. 1c). The ABTS radical scavenging capacity in LvH bran (IC_50_ value of 18.21 mg mL^−1^) was comparable to HongH bran, but significantly lower than GJG bran (0.21-fold), and significantly higher than HeiH bran (3.10-fold) (Fig. 1d). In addition, LvH bran had DPPH radical scavenging capacity with an IC_50_ value of 12.46 mg mL^−1^ DW, which was significantly lower than HongH bran (0.72-fold) and GJG bran (0.35-fold), but significantly higher than HeiH bran (8.41-fold) (Fig. 1e).

### Flavonoid compositions and levels

**Figure 2:**
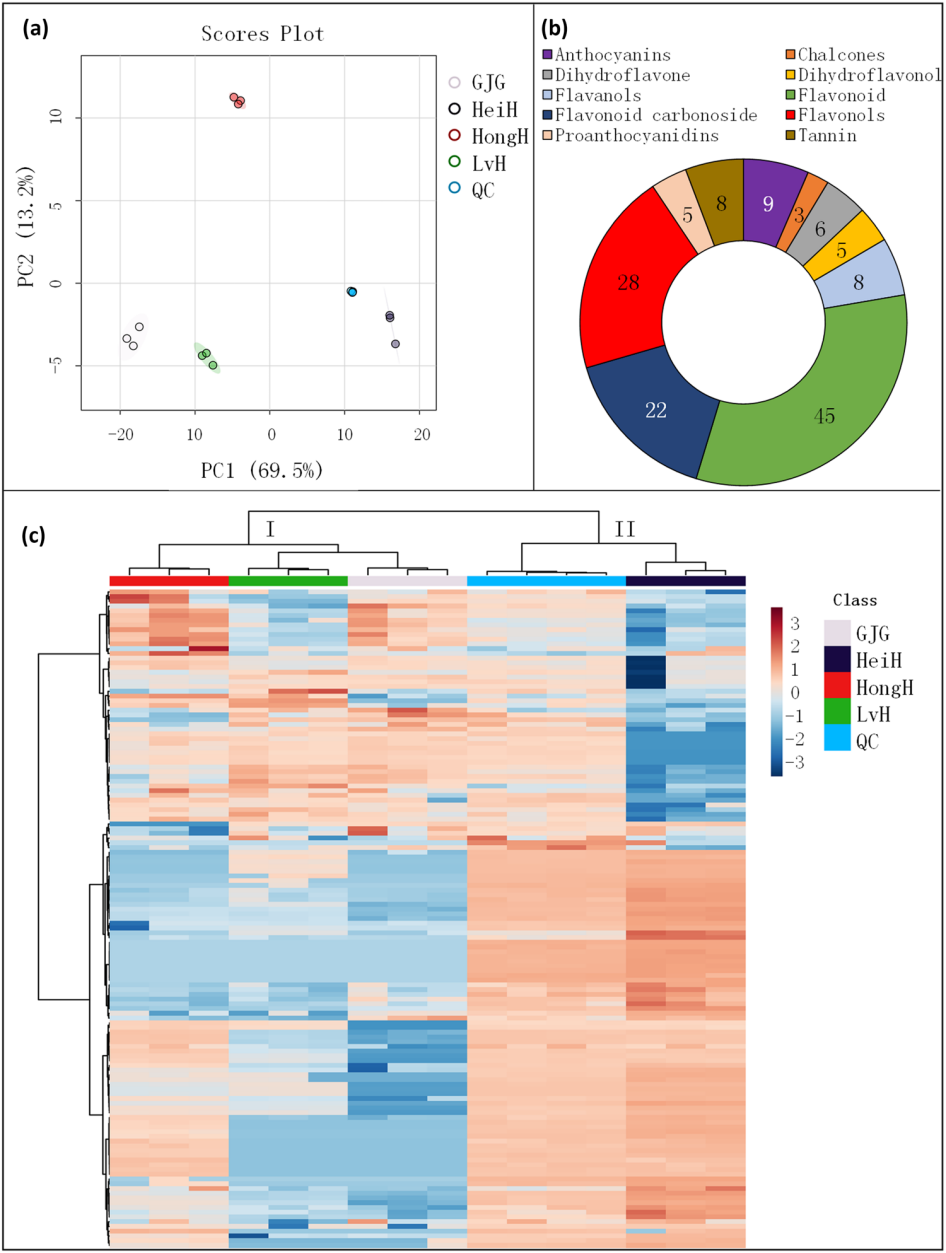
Multivariate statistical analysis of flavonoid metabolites in the bran of LvH, HeiH, HongH and GJG varieties. PCA score (**a**), number of metabolites in groups (**b**), and heatmap (**c**). *PCA* principal component analysis, *QC* quality control.

The flavonoid metabolites in the ethanolic extracts of glutinous rice bran with different colored kernel were analysed using the ultra-performance liquid chromatography coupled to tandem mass spectrometry (UPLC–MS/MS) platform. Principal component analysis (PCA) was used to assess the differences in metabolomes of among various colored rice varieties. The results from the PCA showed distinct separation among the different varieties, as demonstrated by the high PC1 and PC2 values of 69.5% and 13.2%, respectively (Fig. 2a). A total of 139 metabolites were positively identified, including 45 flavonoids, 28 flavonols, 22 flavonoid carbonosides, 9 anthocyanins, 8 flavanols, 8 tannins, 5 proanthocyanidins, 5 dihydroflavonols, and 3 chalcones (Fig. 2b). Apigenin, chrysoeriol, cyanidin, diosmetin, isorhamnetin, kaempferol, luteolin, myricetin, naringenin, orientin, procyanidin, quercetin, syringetin, tricin and their glycosides were abundant, and their glycosides were frequently detected at 3-O, 5-O, 6-C, 7-O, 8-C, and 8-O (Table S1).

**Figure 3:**
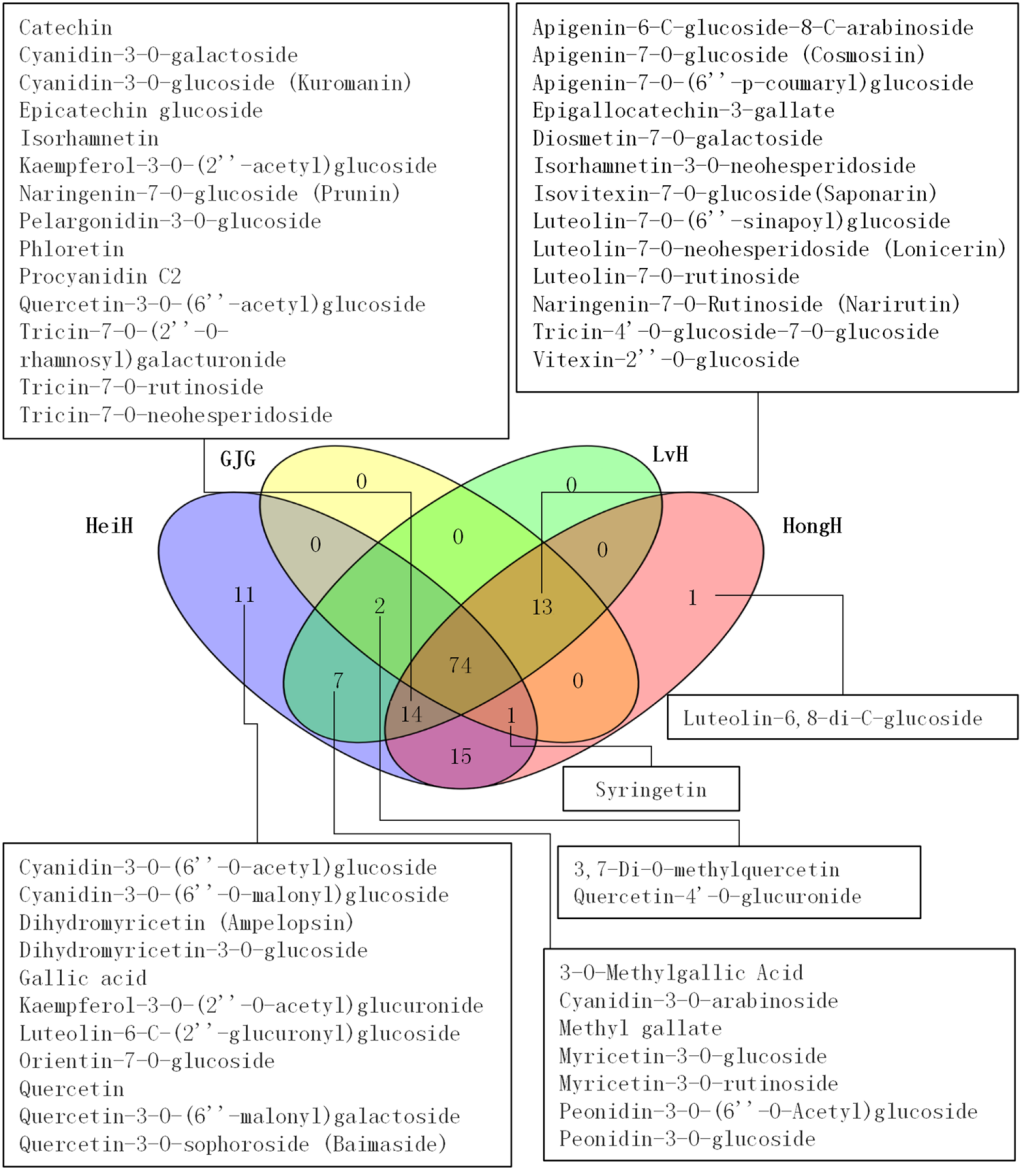
Venn diagram of flavonoid metabolites in the bran layer of LvH, HeiH, HongH and GJG varieties.

The heatmap showed that the levels of metabolites varied greatly among glutinous rice varieties with different kernel colors, and the rice varieties were clustered into 2 subcategories (Fig. 2c). LvH was clustered first with GJG, followed by HongH, while HeiH was clustered individually. Venn diagram of metabolites among glutinous rice varieties was further performed, and the results are presented in Fig. 3. There were 74 overlapping metabolites among all varieties, 11 unique metabolites in HeiH and 1 unique metabolite in HongH, 7 unique metabolites in LvH and HeiH, 13 unique metabolites in LvH, HongH and GJG, 2 unique metabolites in LvH, HeiH and GJG, and 14 unique metabolites in LvH, HeiH and HongH. There were 40, 26, and 22 DMs between the LvH vs. HeiH, LvH vs. HongH, and LvH vs. GJG comparison groups, respectively.

**Figure 4:**
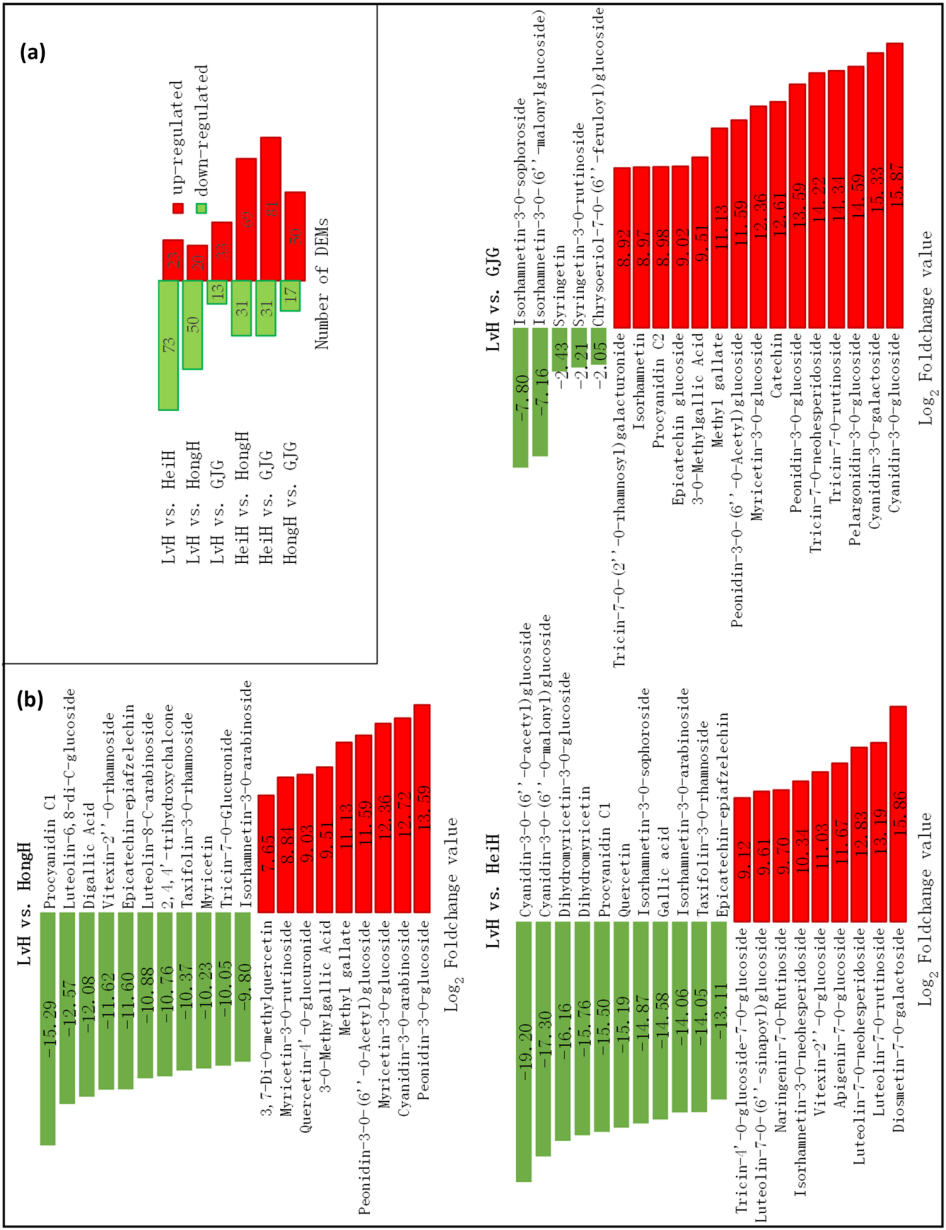
The number of DEMs (**a**) and the top 20 DEMs of fold change (**b**) in the bran layer of LvH, HeiH, HongH and GJG varieties by pairwise analysis.

The number of DEMs between varieties by pairwise comparison is presented in Fig. 4a. LvH had 23, 20 and 33 up-regulated DEMs and 73, 50 and 13 down-regulated DEMs compared to HeiH, HongH and GJG, respectively. The top 20 DEMs of fold change by pairwise comparison are presented in Fig. 4b. Compared to HeiH, LvH contained the top 9 up-regulated DEMs including 8 flavonoid derivatives and 1 dihydroflavone, with Log_2_Fold-change (Log_2_FC) values ranging from 9.12 to 15.86, and the top 11 down-regulated DEMs including 3 dihydroflavonols, 3 flavonols, 2 anthocyanins, 1 flavanol, 1 proanthocyanidin, and 1 tannin, with Log_2_FC values ranging from 13.11 to 19.20. Among them, 7 glycosides at 7-O were up-regulated in LvH and 6 glycosides at 3-O were up-regulated in HeiH. Compared to HongH, LvH contained the 9 top up-regulated DEMs including 3 anthocyanins, 3 flavonols, 2 tannins, and 1 dihydroflavonol, with Log_2_FC values ranging from 7.65 to 13.59, and the 11 top down-regulated DEMs including 4 flavonoids, 2 dihydroflavonol, 1 chalcones, 1 flavanols, 1 flavonols, 1 proanthocyanidin, and 1 tannin, with Log_2_FC values ranging from 9.80 to 15.29. Compared to GJG, LvH contained the top 15 up-regulated DEMs including 5 anthocyanins, 3 flavonoid derivatives, 2 flavanols, 2 flavonols, 2 tannins, and 1 proanthocyanidin, with Log_2_FC values ranging from 8.92 to 15.87, and the top 5 down-regulated DEMs including 3 flavonoid derivatives and 2 flavonols, with Log_2_FC values ranging from 2.05 to 7.80.

### Correlation analysis and identification of candidate biomarkers

**Figure 5:**
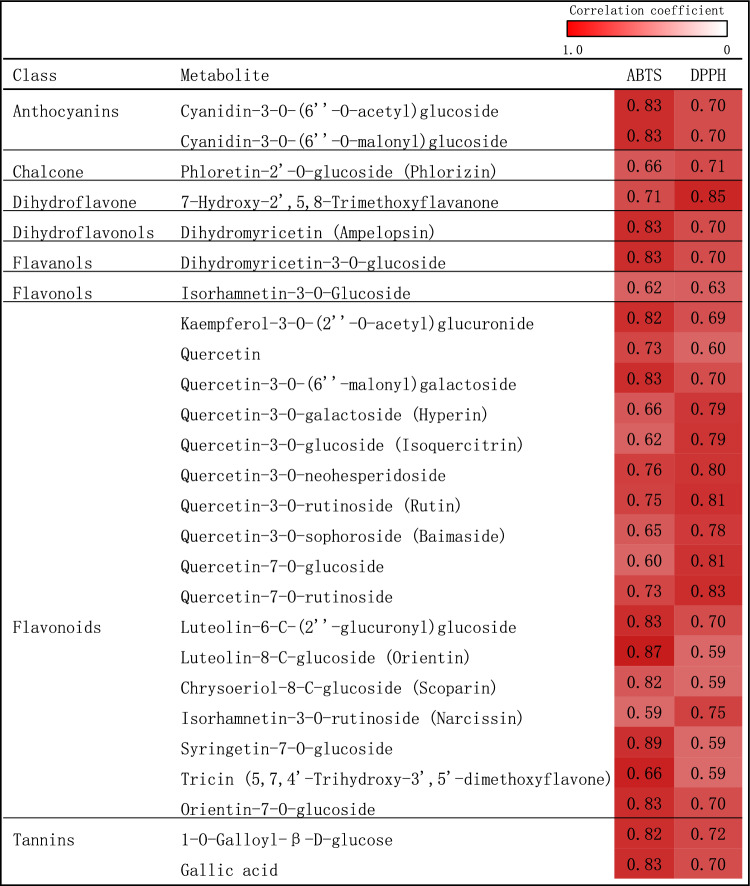
Correlation coefficients between flavonoid metabolites and antioxidant capacity in the bran of LvH, HeiH, HongH and GJG varieties. *DPPH* 1-diphenyl-2-picrylhydrazyl radical scavenging capacity, *ABTS* 2′-azino-bis(3-ethylbenzothiazoline-6-sulfonic acid) radical scavenging capacity.

Correlation coefficients between metabolites and antioxidant capacity are presented in Fig. 5. There were 26 metabolites that were significantly positively correlated with both ABTS and DPPH radical scavenging capacity, such as 9 quercetin derivatives, 2 cyanidin derivatives and 2 dihydromyricetin.

**Figure 6:**
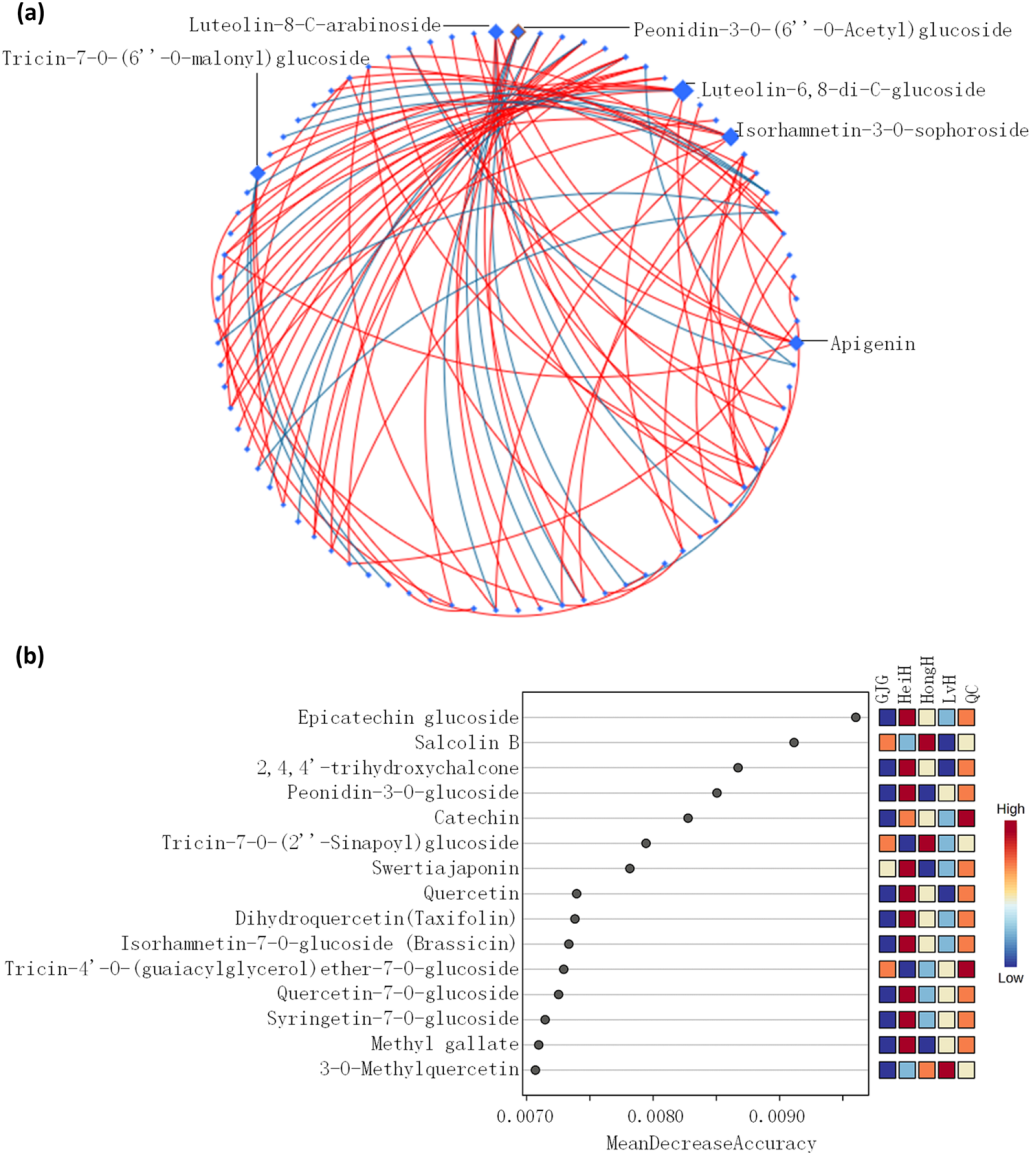
Analysis of flavonoid metabolites in the bran of LvH, HeiH, HongH and GJG varieties by DSPC network (**a**) and Randomforest (**b**).

A visualization of the correlation networks is presented in Fig. 6a. Luteolin-6,8-di-C-glucoside was identified as the core metabolite, followed by luteolin-8-C-arabinoside, peonidin-3-*O*-(6″-*O*-Acetyl)glucoside, isorhamnetin-3-*O*-sophoroside, and apigenin. A total of 15 metabolites were identified as candidate biomarkers based on Randomforest analysis among different colored varieties (Fig. 6b). Epicatechin glucoside was identified as the core metabolite, followed by salcolin B, 2,4,4′-trihydroxychalcone, and peonidin-3-*O*-glucoside.

### Flavonoid metabolic pathway

**Figure 7:**
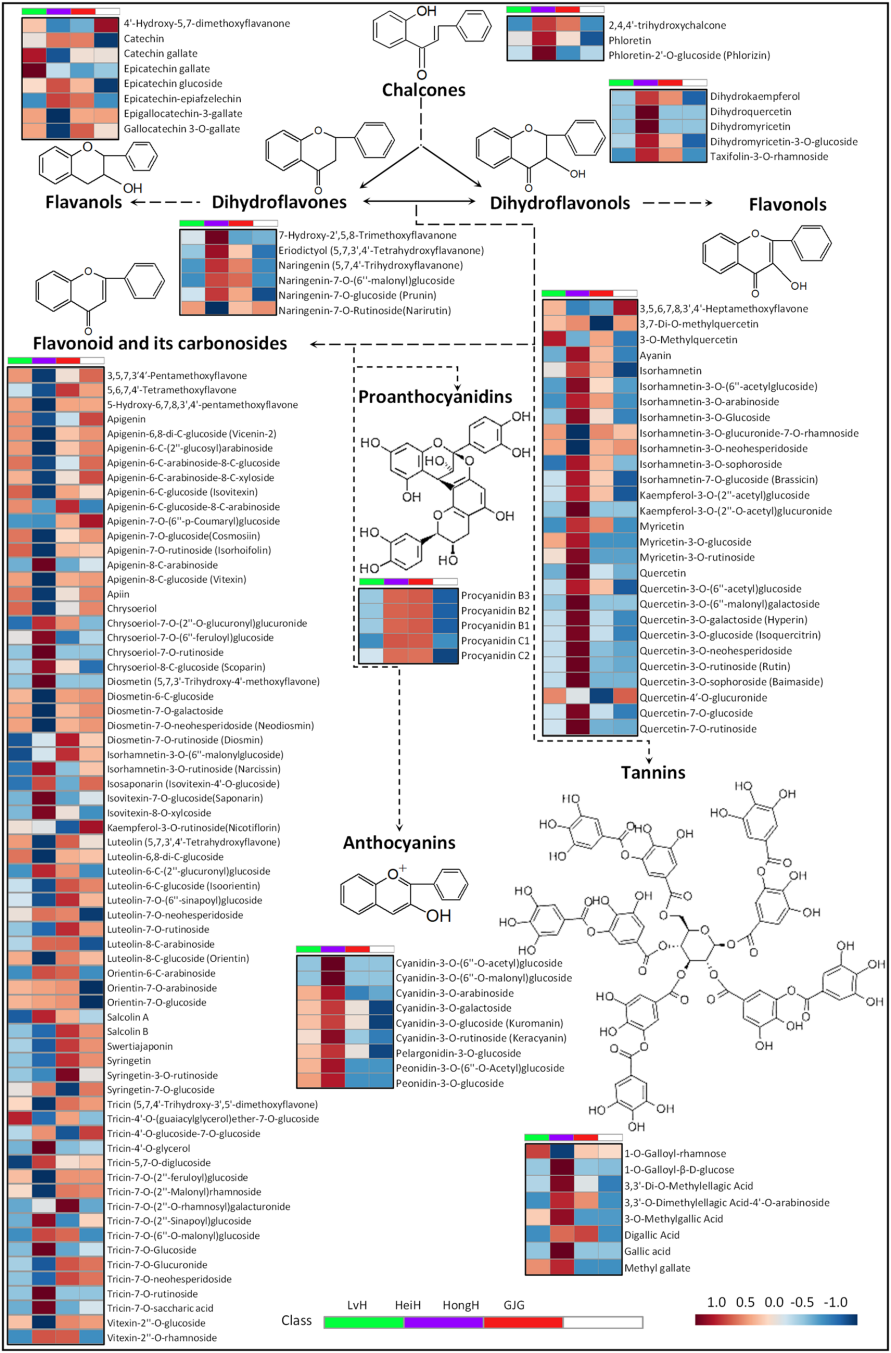
The heatmap of the flavonoid metabolic pathway by comparing flavonoid metabolites in the bran of LvH, HeiH, HongH and GJG varieties.

The heatmap of the flavonoid metabolic pathway is presented in Fig. 7. The levels of metabolites in the flavonoid biosynthesis pathway varied greatly among the different colored varieties. HeiH contained the highest levels of most chalcones, dihydroflavonols and dihydroflavones, followed by HongH, LvH and GJG. Phlorizin, 7-hydroxy-2′,5,8-trimethoxyflavanone, dihydromyricetin, and dihydroquercetin were found at very high levels in HeiH, and 2,4,4′-trihydroxychalcone, dihydrokaempferol, taxifolin-3-*O*-rhamnoside, naringenin, and naringenin-7-*O*-(6″-malonyl)glucoside were found at high levels in HeiH and HongH. HeiH also contained the highest levels of anthocyanidins, followed by LvH, HongH and GJG. Cyanidin-3-*O*-(6″-*O*-acetyl)glucoside, cyanidin-3-*O*-(6″-*O*-malonyl)glucoside, and cyanidin-3-*O*-rutinoside were found at very high levels in HeiH. Cyanidin-3-*O*-arabinoside, cyanidin-3-*O*-galactoside, cyanidin-3-*O*-glucoside, pelargonidin-3-*O*-glucoside, peonidin-3-*O*-(6″-*O*-acetyl)glucoside, and peonidin-3-*O*-glucoside were found at high levels in HeiH and LvH (Fig. 7, Table S1).

Procyanidins were found at high levels in HeiH and HongH, but LvH showed higher levels of 5 procyanidins than GJG. HeiH had very high levels of most flavonols, including ayanin, 6 isorhamnetin derivatives, 2 kaempferol derivatives, 3 myricetin derivatives, and 10 quercetin derivatives. However, 3,5,6,7,8,3′,4′-heptamethoxyflavone, 3-*O*-methylquercetin, isorhamnetin-3-*O*-glucuronide-7-*O*-rhamnoside, isorhamnetin-3-*O*-neohesperidoside, and quercetin-4′-*O*-glucuronide were found at low levels in HeiH, whereas LvH had high levels. HeiH had very high levels of many flavonoids, such as 6 tricin derivatives and 4 chrysoeriol derivatives. However, a total of 36 flavonoid derivatives were found at low levels in HeiH, such as 11 apigenin derivatives, 6 luteolin derivatives, 4 tricin derivatives, and 3 diosmetin derivatives, whereas LvH had high levels in 23 of them. In contrast, LvH showed similar levels of many flavonoid derivatives compared to HongH and GJG.

## Discussion

Rice bran contains a high fiber content and shows a rapid development of rancidity, making it unpalatable for human consumption. However, rice bran is a valuable source of bioactive compounds and has been recognized as a potential provider of antioxidants^1,2^. It has been reported that the addition of 30% rice bran to wheat bread results in a five-fold improvement in its antioxidant potential^33^. The antioxidant capacity of plants is generally associated with the intensity of their coloration^11^. It is well-established that purple and red colored rice exhibit stronger antioxidant capacity in their bran compared to white rice^11,12^. In this study, consistent results were obtained, showing that the bran of purple (HeiH) and red (HongH) glutinous rice had higher radical scavenging capacity of DPPH and ABTS compared to white glutinous rice (GJG). However, green kernel of rice remains a rare germplasm worldwide^32^. LvH showed light green kernel with taupe grain, making it easily distinguishable from commonly found colored rice varieties such as purple, red and white rice. Moreover, LvH bran demonstrated significantly higher radical scavenging capacity of DPPH not only compared to GJG but also HongH, suggesting its potential as a valuable source of antioxidants.

Flavonoids, primarily stored in the bran layer of cereals, are important antioxidants with effective health-promoting activities^9,34^. It has been reported that the TFC of 178 wheat germplasms (6 black, 24 red, and 148 white) ranged from 0.147 to 0.397 mg g^−1^, with an average TFC of black, red, and white wheat germplasms being 0.361 mg g^−1^, 0.290 mg g^−1^, 0.241 mg g^−1^, respectively^35^. By contrast, the TFC of 481 rice germplasms (6 black, 52 red, and 423 white) ranged from 0.886 to 2.863 mg g^−1^, with an average TFC of black, red, and white rice germplasms being 2.406 mg g^−1^, 1.472 mg g^−1^, 1.316 mg g^−1^, respectively^36^. In this study, LvH bran contained significantly higher TFC than that of HongH (1.91-fold) and GJG (2.45-fold). Besides, the IC_50_ value of DPPH radical scavenging capacity in LvH bran was even lower than that of HongH bran, suggesting that the bran of green glutinous rice could be used as a potential natural flavonoids source with high antioxidant capacity.

Flavonoids are also the predominant pigment metabolites in the bran layer of rice^9^. Through the UPLC–MS/MS platform, a total of 139 flavonoid metabolites in the bran layer of four different colored glutinous rice were identified. Among these metabolites, anthocyanins play a major role in determining the color of rice kernels^37^. The bran of HeiH and LvH contained derivatives of cyanidin, peonidin, and pelargonidin by UPLC–MS/MS analysis, whereas HongH lacked peonidin, and GJG lacked both peonidin and pelargonidin. These DEMs of anthocyanins among rice varieties probably contributed to the distinct coloration of rice kernels. Besides, anthocyanins, being potent antioxidants found in the bran of colored rice cultivars, offer various health benefits, including anti-aging, anti-inflammatory, and antiviral activities^38^. Colored rice varieties, especially purple and red rice, possess significantly higher anthocyanin content than white rice, making them a recognized source of natural anthocyanins^37,38^. It was noteworthy that the TAC of LvH bran was not only significantly higher than that of white rice GJG (13.30-fold), but also several times higher than that of red rice HongH (4.34-fold), indicating that green glutinous rice was also a valuable natural source of anthocyanin.

Colored rice contains many dominant antioxidants, such as apigenin, cyanidin, isorhamnetin, kaempferol, luteolin, myricetin, quercetin, and tricin, which are frequently detected as O- or C-glycosides^9^. The flavonoids with multiple hydroxyl substitutions have stronger antiperoxyl radical activities^13^. It has been reported that eupatilin, diosmin, diosmetin, l-glutamic acid, pantothenic acid and pebrellin are candidate metabolite markers for screening antioxidant capacity in purple glutinous rice^17^. Significantly positive correlations were identified between 26 metabolites and the radical scavenging capacity of both ABTS and DPPH, including 11 flavonols, 7 flavonoids, 2 anthocyanins, 2 flavanols, 2 tannin derivatives, 1 chalcones, 1 dihydroflavone, and 1 dihydroflavonol. Notably, quercetin derivatives were the substances with the highest number of correlations with antioxidant activity, followed by cyanidin derivatives and luteolin derivatives. Quercetin is an excellent natural antioxidant with multiple biological activities^39,40^. Therefore, the levels of quercetin derivatives were proposed as a potential indicator for assessing the antioxidant capacity of different colored rice bran. A significant and robust relationship (edge) was established by using a large-scale metabolomics data analysis tool to draw the debiased sparse partial correlation (DSPC) network of metabolites^41^, and luteolin-6,8-di-C-glucoside was identified as the core metabolite, which could provide a reference for a better understanding of the relationships between flavonoid metabolites. In addition, candidate biomarkers were screened based on Randomforest analysis, and epicatechin glucoside was identified as the core candidate biomarker.

Metabolomics is a powerful tool for detecting metabolites within metabolic pathways, revealing distinct differences in flavonoid metabolite synthesis among colored rice varieties^42^.Using the UPLC–MS/MS platform, LvH bran was found to typically have elevated levels of apigenin derivatives, catechin gallate, diosmetin derivatives, epicatechin gallate, and tricin-4′-*O*-(guaiacylglycerol)ether-7-*O*-glucoside compared to HeiH, HongH, and GJG. In addition, LvH bran had significantly higher levels of 23 flavonoids that were present at low levels in HeiH bran. Notably, LvH exhibited elevated levels of several flavonol and flavonoid metabolites compared to GJG. However, LvH displayed lower levels of many flavonol and flavonoid metabolites when compared to HongH. These findings indicated considerable disparities in downstream metabolite synthesis in the flavonoid pathway among different colored glutinous rice varieties, particularly between purple and green glutinous rice.

In conclusion, the bran of green glutinous rice (LvH) had significantly higher TFC and TAC than red (HongH) and white (GJG) glutinous rice. Additionally, LvH bran had significantly higher vitamin B1 and vitamin E than purple (HeiH) and red (HongH) glutinous rice (Fig. S1), demonstrating green glutinous rice bran was rich in bioactive components. LvH bran showed significantly lower IC_50_ values for scavenging DPPH and ATBS radicals compared to GJG, and even exhibited significantly lower IC_50_ value for scavenging DPPH radicals compared to HongH, highlighting its potential as an effective source of antioxidants. LvH bran contained derivatives of cyanidin, peonidin, and pelargonidin but at lower levels than HeiH, whereas HongH bran lacked peonidin, and GJG bran lacked both peonidin and pelargonidin, which might explain variations in rice kernel colouration. There were 26 metabolites that showed a significant positive correlation with both ABTS and DPPH radical scavenging capacity. Quercetin derivatives were found to be the most correlated substances with antioxidant activity, possibly indicating their potential as useful indicators for evaluating the antioxidant capacity of different colored rice bran. Through the UPLC–MS/MS platform, LvH bran had 40, 26, and 22 DMs, 23, 20, and 33 up-regulated DEMs, and 73, 50, and 13 down-regulated DEMs compared to HeiH, HongH, and GJG, respectively, indicating significant differences in downstream metabolite synthesis of flavonoid pathway among various colored glutinous rice cultivars, with a particular distinction between green and purple varieties. Notably, LvH bran had elevated levels of apigenin derivatives, catechin gallate, diosmetin derivatives, epicatechin gallate, and tricin-4′-*O*-(guaiacylglycerol)ether-7-*O*-glucoside. In addition, the DSPC metabolite network revealed luteolin-6,8-di-C-glucoside as the core metabolite. Epicatechin glucoside was identified as the core candidate biomarker through Randomforest analysis. These findings provide valuable insights into the antioxidant properties of green glutinous rice bran and serve as references for gaining a better comprehension of flavonoid metabolites in different colored rice bran.

## Materials and methods

### Plant materials

Green variety ‘Lvhe’ (LvH), purple variety ‘Heihe’ (HeiH), red variety ‘Honghe’ (HongH), and white variety ‘Goujingao’ (GJG) of KSR were planted at the Experimental Station of the Institute of Plant Genetics and Breeding at Guizhou Normal University, China (1146.0 m, 26° 50′ N, 106° 58′ E). The randomized blocks design was performed with 3 replications. Rice grains were harvested at maturity and dried naturally to 14.0% of the grain water content. Then, rice grain was processed into brown rice by a JLGJ-45 power rice huller, and the bran layer was ground and collected by a JNMJ-3 rice milling machine with three biological replicates.

### Determination of TFC and TAC

The bran layer powder was previously sifted using a 100-mesh sieve. A total of 100 mg sample was extracted using 2 mL of 80% ethanol in a 70 °C for 6 h, followed by 10 min of ultrasound-assisted extraction. The supernatant was then collected to measure the TFC according to the method of Wang^43^. A total of 50 mg sample was extracted using 2 mL of 0.1 mol L^−1^ HCl at 32 °C for 1 h, followed by 10 min of ultrasound-assisted extraction. The TAC was measured according to the method of Giusti^44^.

### Determination of vitamin B1 and vitamin E

A total of 100 mg sample was added to 0.5 mL of 0.01 mol L^−1^ HCl and homogenized, followed by 30 min of ultrasound-assisted extraction. The mixture was then filtered using a needle filter to determine vitamine B1 according to the instructions of VB1-1-G kit (Suzhou Keming Biotechnology Co., Ltd, Suzhou, China). A total of 100 mg sample was added to 1 mL of VE-1-G kit extraction solution and homogenized, followed by 5 min shaking using a vortex mixer. The mixture was then centrifuged at 5000*g* for 10 min at 25 °C. The supernatant was used to determine vitamine E according to the instructions of VE-1-G kit (Suzhou Keming Biotechnology Co., Ltd, Suzhou, China).

### Determination of antioxidant activities

A total of 100 mg sample was added to 1 mL of DPPH-1-D kit extraction solution and homogenized in an ice bath. The mixture was then centrifuged at 10,000*g* for 10 min at 4 °C. The supernatant was diluted to different concentrations to determine the IC_50_ value of DPPH radical scavenging capacity according to the instructions of DPPH-1-D kit (Suzhou Keming Biotechnology Co., Ltd, Suzhou, China). A total of 100 mg sample was added to 1 mL of ABTS-1-D kit extraction solution and homogenized in an ice bath. The mixture was then centrifuged at 10000*g* for 10 min at 4 °C. The supernatant was diluted to different concentrations to determine the IC_50_ value of ABTS radical scavenging capacity according to the instructions of ABTS-1-D kit (Suzhou Keming Biotechnology Co., Ltd, Suzhou, China).

### UPLC–ESI–MS/MS analysis

Samples were lyophilized by vacuum freeze dryer (Scientz-100F) and crushed by mixer mill (MM 400, Retsch) with zirconia beads for 1.5 min at 30 Hz. Then, 100 mg of lyophilized powder was dissolved in 1.2 mL 70% methanol solution by oscillating 30 s every 30 min 6 times. The samples were centrifuged at 12,000 rpm for 10 min after being stored in a refrigerator at 4 °C overnight, and the extracts were filtered (SCAA-104, pore size 0.22 μm; ANPEL, Shanghai, China). The UPLC–ESI‒MS/MS system (UPLC, SHIMADZU Nexera X2, MS, Applied Biosystems 4500 Q TRAP) was performed using a UPLC column (Agilent SB-C18, 1.8 µm, 2.1 mm*100 mm) with solvent A (pure water with 0.1% formic acid) and solvent B (acetonitrile with 0.1% formic acid) by 0.35 mL per minute of flow velocity, 40 °C of column oven, 4 μL of injection volume, and following the operating steps: a linear gradient from 5 to 95% of solvent B was performed within 9 min and kept for 1 min, then decreased to 5% in 1.10 min and kept for 2.9 min.

### Data analysis

Data were collected by a triple quadrupole-linear ion trap mass spectrometer (Q TRAP), AB4500 Q TRAP UPLC/MS/MS system equipped with an ESI Turbo Ion-Spray interface operating in positive and negative ion mode and controlled by Analyst 1.6.3 software (AB Sciex). The parameters were set as follows: source temperature, 550 °C; ion spray voltage, 5500 V (positive)/− 4500 V (negative); ion source gas I, 50 psi; gas II, 60 psi; curtain gas, 25.0 psi; and high collision-activated dissociation.

Multivariate data analysis was performed using the R package ropls. Unsupervised PCA was performed using the base package of R (Version 3.5.0, www.r-project.org). OPLS-DA was performed by MetaboAnalystR of R (Version 1.0.1). The heatmap was generated using the pheatmap of R (V1.0.12). DEM screening (fold change ≥ 2 or fold change ≤ 0.5, VIP ≥ 1.0 and *p* value < 0.05) was performed. The heatmap was created using the cloud platform of MetaboAnalyst (https://new.metaboanalyst.ca/MetaboAnalyst). The Kyoto Encyclopedia of Genes and Genomes (KEGG) analysis of DEMs was performed using the cloud platform of database (http://www.kegg.jp/kegg/kegg1.html). Venn diagram was performed using the cloud platform of Omicstudio (https://www.omicstudio.cn/tool/43). The Pearson correlation coefficient was determined using the cloud platform of MetaboAnalyst (https://new.metaboanalyst.ca/MetaboAnalyst).

Raw data of physiological active components and antioxidant capacity were organized using WPS software (Kingsoft Office, version 2019, China) and analysed by one-way ANOVA using SPSS software (IBM, version 19.0, United States).

### Ethical statement

The permission of this study was obtained from Institution of Plant Genetics and Breeding, Guizhou Normal University. The experimental research and field studies on plants (either cultivated or wild) in this study were complied with relevant institutional, national, and international guidelines and legislation.

### Supplementary Information

Supplementary Table S1.

Supplementary Figure S1.

## Data Availability

The datasets used and/or analysed during the current study available from the corresponding author on reasonable request.
