# Supplementary figures and images for: Characteristics of antioxidant capacity and metabolomics analysis of flavonoids in the bran layer of green glutinous rice (Oryza sativa L. var. Glutinosa Matsum)
Source: Sci Rep. 2023 Sep 29;13:16372. doi: 10.1038/s41598-023-43466-3 (PMC10541414; doi:10.1038/s41598-023-43466-3)

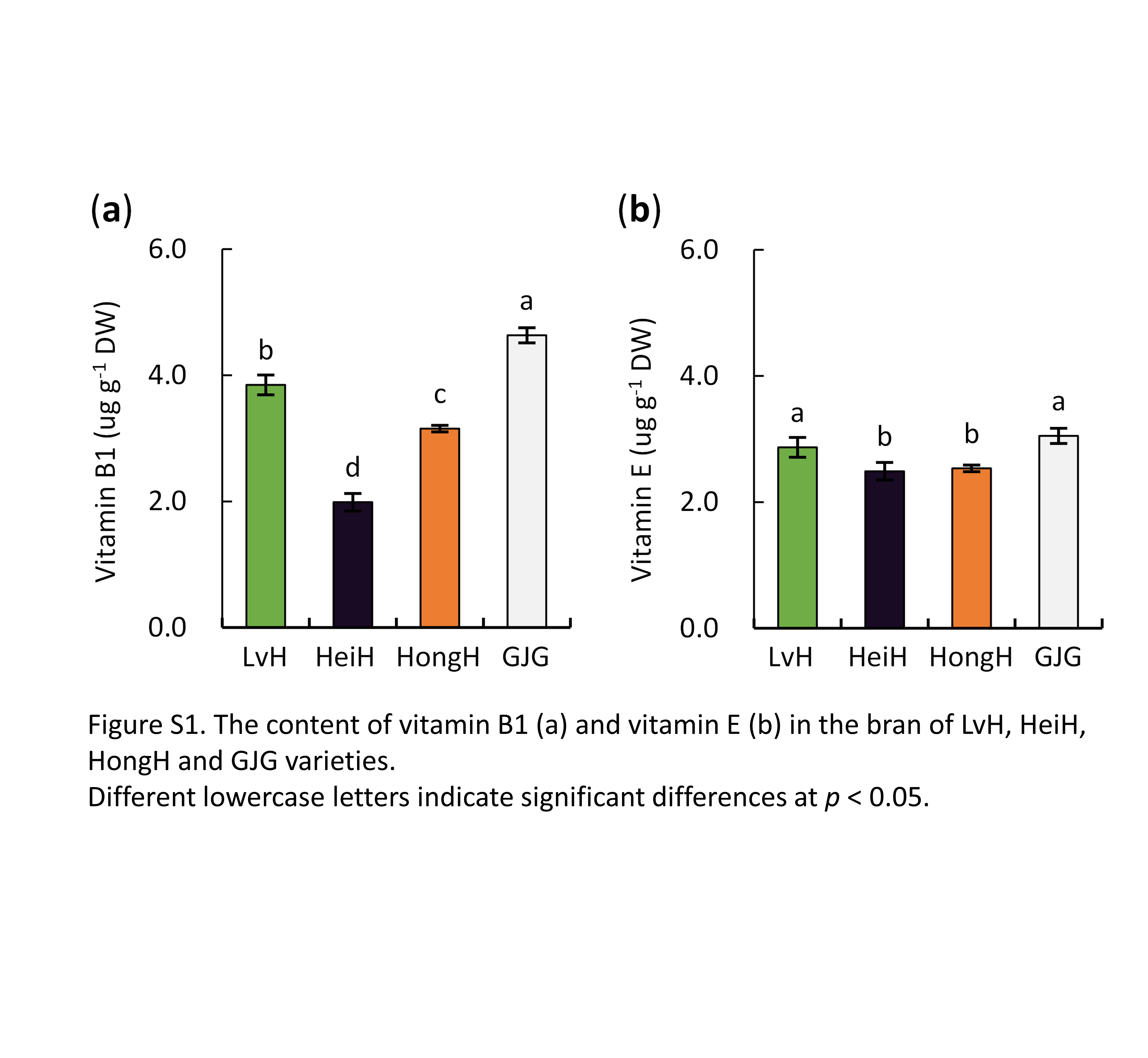

Supplement: Supplementary file 2 — Supplementary Figure S1. [file 41598_2023_43466_MOESM2_ESM.tif]
